# Here Today, Gone Tomorrow – Adaptation to Change in Memory-Guided Visual Search

**DOI:** 10.1371/journal.pone.0059466

**Published:** 2013-03-15

**Authors:** Martina Zellin, Markus Conci, Adrian von Mühlenen, Hermann J. Müller

**Affiliations:** 1 Department Psychologie, Ludwig-Maximilians-Universität München, München, Germany; 2 Department of Psychology, University of Warwick, Coventry, United Kingdom; University of Groningen, The Netherlands

## Abstract

Visual search for a target object can be facilitated by the repeated presentation of an invariant configuration of nontargets (‘contextual cueing’). Here, we tested adaptation of learned contextual associations after a sudden, but permanent, relocation of the target. After an initial learning phase targets were relocated within their invariant contexts and repeatedly presented at new locations, before they returned to the initial locations. Contextual cueing for relocated targets was neither observed after numerous presentations nor after insertion of an overnight break. Further experiments investigated whether learning of additional, previously unseen context-target configurations is comparable to adaptation of existing contextual associations to change. In contrast to the lack of adaptation to changed target locations, contextual cueing developed for additional invariant configurations under identical training conditions. Moreover, across all experiments, presenting relocated targets or additional contexts did not interfere with contextual cueing of initially learned invariant configurations. Overall, the adaptation of contextual memory to changed target locations was severely constrained and unsuccessful in comparison to learning of an additional set of contexts, which suggests that contextual cueing facilitates search for only one repeated target location.

## Introduction

Experience greatly influences our perception of the visual world. For example, familiar contingencies between scenes and objects can support target identification (see [1] for review). Specifically, observers can identify a loaf of bread faster than a similarly shaped post box both presented in the same kitchen scene [2]. Such observations suggest that processing of target objects benefits from a coherent and familiar scene context. Although natural scenes may remain quite stable, observers are often required to detect a target object placed at changing locations. For example, in an otherwise invariant kitchen scene, a saucepan can sometimes be located on the stove and at other times on the table. If observers are familiar with the kitchen scene, they will find the saucepan relatively quickly, irrespective of its variable location. By contrast, other objects, such as a kettle, usually stay in one place in the kitchen, and if they are moved to a new position, the relocation will be relatively permanent. In order to ensure quick search for such permanently relocated targets, context-target associations would have to be adapted to the new situation in the longer term; that is, already established representations would have to be relearned.

In the present study, we investigated whether observers can adapt memory representations of context-target associations when targets are relocated permanently within their contexts (relearning). We further distinguished relearning as one kind of memory adaptation from adapting to entirely new contextual relations (new-learning). For example, new-learning is required when visual search is performed in a further kitchen scene after having successfully learned an initial kitchen scene.

**Figure 1:**
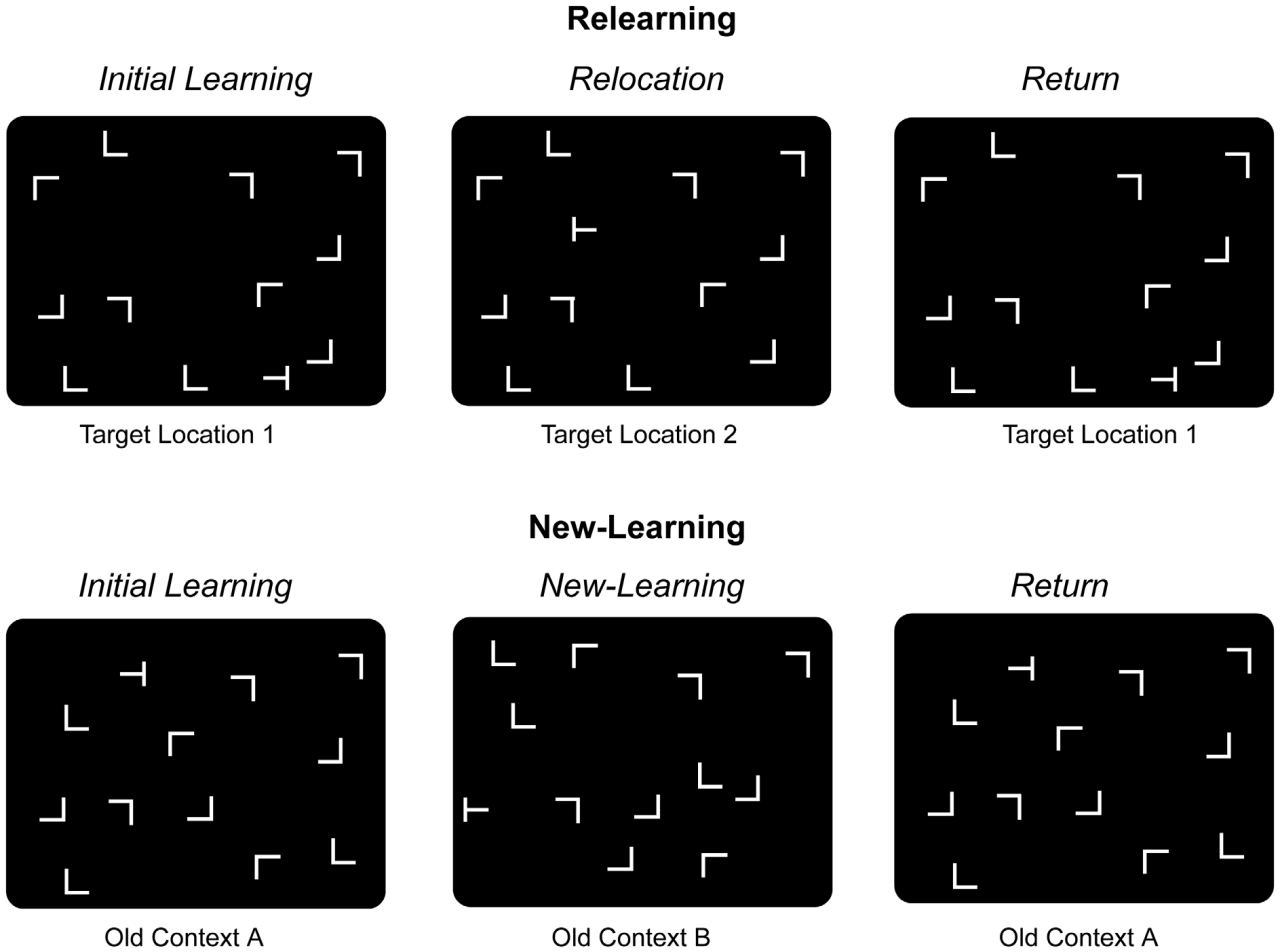
Example search displays and procedure for relearning and new-learning experiments. Relearning (Experiments 1 and 3): (Top Half) Old-context displays were presented with initial target locations in a first learning phase (Target Location 1). Then the relocation phase followed, presenting the targets repeatedly at novel, previously empty positions in otherwise unchanged old contexts (Target Location 2), requiring observers to relearn previous contextual associations. In a final return phase the initial target locations (Target Location 1) were presented again (Experiments 1A and 3 only). New-learning (Experiments 2 and 4): (Bottom Half) After an initial learning phase (Old Context A), an additional set of distinct old-context displays was repeatedly presented (Old Context B) to instigate new-learning of contextual associations. Old context displays from the learning phase (Old Context A) were presented again in a final return phase (Experiments 2A and 4 only).

To examine relearning of spatial representations as well as new-learning, observers learned spatial contingencies between a context and a target location within the contextual cueing paradigm (see [3] for review). In a typical experiment [4], observers searched for a target letter ‘T’ amongst a configuration of eleven nontarget ‘Ls’ (see Figure 1). Unknown to the observers, some of the search displays were repeatedly presented with invariant spatial layouts of the targets and nontargets (old contexts) throughout the experiment. Results showed faster response times (RTs) to old contexts than to randomly generated new contexts (the ‘contextual-cueing effect’). In a subsequent recognition test observers were unable to discern old from new displays ([5], but see [6]), suggesting that observers implicitly learned to associate an old configuration of nontargets with a target location, guiding visual search more efficiently to the target object.

Before considering the rationale of our study, we first review previous findings on contextual cueing of two variable target locations (e.g., a saucepan placed at variable locations within a kitchen context). Next, we summarize studies that investigated relearning of a second, permanently relocated target (e.g., a permanently relocated kettle in a kitchen). Finally, as a comparison, we present studies on sequential new-learning; that is, learning of sequentially presented distinct sets of context-target configurations.

### Multiple Target Locations in Contextual Cueing

In agreement with observers’ ability to detect targets efficiently at variable locations in natural scenes, Chun and Jiang (1998, [4]) reported that old contexts may be associated with at least two target locations. In Experiment 6 of their study, contexts were repeatedly presented with two different target locations (on separate, randomly selected trials), which yielded reliable, but reduced contextual cueing [7] in comparison to contexts that were associated with only one target location (in different experiments). Brady and Chun (2007, [8]) explained this observation in terms of a (computational) model, which assumes that each encounter with an invariant context-target pairing strengthens the association between a target location and its local context of a few neighboring items (see [9] for a comparable neurophysiological model of contextual cueing). Based on this assumption, the model predicts that repeated visual search for two target locations in the same context results in contextual cueing for both locations (*multiple-target learning*). The model also predicts that the simultaneous cueing of both learned locations would slow target detection because both target locations compete with each other for focal-attentional selection. Consequently, the average cueing effect of the respective context would be reduced compared with contexts containing only one target location [4].

By contrast, Zellin, Conci, von Mühlenen and Müller (2011, [10]) argued that the integration of multiple target locations into one invariant context is rather unlikely. Similar to [4], two (or three) different target locations were presented within a given invariant context in alternating order, in separate consecutive trials. This ensured that the context was equally predictive of each target location. Overall, contextual cueing was reduced for contexts with two target locations in comparison to contexts with one location, consistent with previous findings [4], [7], [8]. However, more detailed analyses revealed that the overall reduction in contextual cueing was due to an RT-benefit for only one “dominant” of the two (or three) repeated target locations (*single-target learning*). Thus, reduced contextual cueing for contexts with two target locations [4], [7], [8] resulted from averaging across a cued and an uncued target location. Overall, the results of [10] suggest that the memory representations underlying contextual cueing are rather inflexible with regard to accommodating more than one target location.

While multiple-target learning would effectively reduce the average contextual-cueing effect [8], single-target learning maintains efficient visual search for at least one repeated target location [10]. Therefore, single-target learning is advantageous when a target object appears rather unpredictably at different locations (as in the studies discussed above). However, if a target is permanently relocated within its invariant context, the context becomes a reliable cue for this new target location – hence, the respective context-target associations should be relearned to include the contextually new, highly relevant object [11].

### Relearning in Contextual Cueing

A number of studies have demonstrated that target relocations impair contextual cueing of the respective contexts [4], [12] and that relearning might not occur when a learned context becomes associated with a new target location [13], [14]. For example, in the study of Manginelli and Pollmann (2009, [12]), after initial target locations had been presented repeatedly within invariant contexts, the targets were suddenly relocated to new, formerly empty locations within the same contexts of nontargets. While contextual cueing was observed for initial target locations, cueing did not develop for relocated targets despite repeated presentations at the new positions (see also [13], [14]; see top half of Figure 1 for an example display). Rather, search behavior, as assessed by RTs, was comparable to search in new-context displays – which suggests that search was not systematically (mis-)guided to the initial target locations, either [10]. Possibly, the lack of relearning was due to rather short training periods with relocated targets. In sum, to our knowledge, there are no studies that show successful adaptation of existing contextual representations to changed target locations.

### New-Learning in Contextual Cueing

Relearning of new (permanent) target locations in previously learned contexts seems rather improbable in contextual cueing [13], [14]. This lack of relearning could imply general restrictions on learning novel contextual information. Rates of new-learning are usually investigated by conducting two contextual cueing experiments sequentially. That is, after an initial learning phase containing a first set of old contexts further invariant contexts are repeatedly presented (see Figure 1, bottom half, for example displays). Using this experimental approach, Mednick, Makovski, Cai, and Jiang (2009, [15]) showed that reliable new-learning of a second set of old contexts occurred when observers rested or slept before the new-learning phase, but not when they were awake. Similarly, successful new-learning was reported for multiple sets of old contexts when they were presented on separate, consecutive days [16]. In both studies [15], [16], the authors argued that, in principle, active old memory representations from initial learning experiences could proactively interfere with subsequent contextual learning. Hence, following initial contextual learning, additional learning of further old contexts is compromised to some extent. However, proactive interference may be effectively reduced by sleep, as indicated by the results of both studies. Consequently, learning effects can occur in an additional second learning phase if observers sleep after initial learning.

Further studies on other tasks of implicit learning suggest that new-learning (without breaks) may already be facilitated by intense training with the second set of to-be-learned material (statistical learning [17]; implicit motor learning [18]) – though, to our knowledge, this has not been tested for contextual cueing. Although new-learning has been observed in previous studies on implicit learning [15]–[18], initial learning was usually more reliable than new-learning [15], [17], [18]. This primacy effect [17], [18] probably resulted from proactive interference exerted by initial learning experiences over subsequent learning. In sum, a number of studies have demonstrated that implicit new-learning can develop under specific circumstances such as intensive training and after sleep breaks.

### The Present Study

Several studies have shown that contextual relearning of new target locations is inefficient [12]–[14]. By contrast, new-learning of further contexts appears to occur reliably under specific training conditions [15], [16]. The present study was designed to systematically investigate differences between memory adaptation in relearning and in new-learning by testing both under identical experimental conditions. First, similar experimental phases were used to examine relearning (Experiment 1 & 3) and new-learning (Experiment 2 & 4; see top and bottom half of Figure 1 for an example of the experimental phases). Observers learned old contexts in an *initial learning phase*. Then, targets were relocated to new, formerly empty positions in relearning experiments (*relocation phase*), while a further set of previously unseen contexts was presented to observers in new-learning experiments (*new-learning phase*). In particular, in relocation experiments, change occurred in terms of relocated targets presented in (unchanged) old contexts. By contrast, in new-learning experiments, change was implemented by introducing newly arranged and previously unseen old context-target layouts. In four experiments (Experiment 1A, 2A, 3, & 4) old contexts from the initial learning phases were again presented in a final experimental phase (*return phase*).

Second, relearning and new-learning were tested under identical training conditions that had previously been reported to promote successive implicit learning [15]–[18]. Because associations between target locations and surrounding nontarget configurations are consolidated by repeated encounters ([15], [19]; see also [20], [21], for different implicit learning tasks), the initial learning phase was quite short, which might facilitate relearning due to relatively unconsolidated initial associations [22]. At the same time, the subsequent relocation phase was much longer than in previous studies (e.g., at least twice as long as in [12]). Two different lengths of the relocation phase were implemented to examine whether training with relocated targets would eventually result in successful relearning. In addition, in a further experiment, an overnight break separated initial learning from the relocation phase to test whether sleep would reduce proactive interference and, thus, enable contextual relearning [15], [16]. The same training conditions were used for new-learning experiments. Finally, the return phase tested whether the presentation of relocated targets or further old contexts would affect contextual cueing for old context-target layouts from the initial learning phase.

New-learning should occur when observers are intensely trained with the second set of old contexts [17], and new-learning should be particularly effective after extended breaks including sleep [16]. Relearning changed target locations should occur under identical training conditions, if adaptation to relocated targets involves the acquisition of new contextual information in a similar manner as new-learning. In this view, repeated search for relocated targets would eventually lead to successful relearning, and maybe even to contextual cueing of both initial and relocated targets (multiple-target learning [8]). On the other hand, relearning might be rather restricted (single-target learning [10]), preventing contextual cueing of a second target location.

## Experiment 1

Experiment 1 was designed to test whether intensive training promotes relearning of relocated targets. In Experiment 1A, old-context displays were repeatedly paired with initial target locations in a learning phase (15 presentations). Subsequently, targets were relocated to new, formerly empty positions (relocation phase) and repeatedly presented (20 presentations). Following the relocation phase, initial target locations returned for another 5 presentations (return phase; see top half of Figure 1 for an example sequence). Experiment 1B was similar to Experiment 1A, except that the relocation phase was further extended (to 35 presentations) and the return phase was abolished. The latter was done to enable longer training, while ensuring a reasonable total duration of the experiment.

### Method

#### Observers

In Experiment 1A, 12 adults (10 women) were tested. Mean age was 23.3 years (range: 19–31 years). Another 12 adults (9 women) took part in Experiment 1B, with a mean age of 24.4 years (range: 21–30 years). All observers reported normal or corrected-to-normal visual acuity and were right-handed. They received either payment (8 or 10 €) or course credits for their participation. As in a previous study [13], only observers who showed positive, larger-than-zero contextual-cueing effects (RT(new)-RT(old)) in the initial learning phase were included in the main analysis because the current study aimed to investigate how changes of the target location affected existing contextual associations. By definition, observers who failed to show contextual cueing for old contexts in the first part of the experiment cannot contribute to answering this question (see Analysis of Excluded Observers for details). The same procedure was adopted in all other experiments reported below (see also [7], [23]–[25] for a comparable procedure).

#### Ethics statement

The ethics board of the Department of Psychology at Ludwig-Maximilians-Universität in Munich approved the present study and its consent procedure before conducting the experiments. The experimental procedure was designed according to the guideline of the Declaration of Helsinki. Observers were comprehensively informed about the study and their rights and provided informed consent before any experiment started. Because the study was non-invasive and all data were processed anonymously, observers were asked to only give verbal consent.

#### Apparatus and stimuli

Stimulus presentation and response collection was controlled by an IBM-PC compatible computer using Matlab Routines and Psychophysics Toolbox extensions [26], [27]. A standard mouse was used as the response device. Stimuli subtended 0.7° x 0.7° of visual angle and were presented in gray (8.5 cd/m^2^) against a black background (0.02 cd/m^2^) on a 17″ CRT monitor. Search displays consisted of 12 items, one of which was a T-shaped target rotated randomly by 90° either to the left or right. The eleven remaining items were L-shaped nontargets rotated randomly in one of the four orthogonal orientations. Search displays were generated by placing the targets and nontargets randomly in the cells of a 6×8 matrix, with an individual cell size of 2.5° x 2.5°. Nontargets were jittered horizontally and vertically in steps of 0.1°, within a range of ±0.6°. Example search displays are shown in Figure 1 (top half). Participants were seated in a dimly lit room with an unrestrained viewing distance of approximately 57 cm from the computer screen.

#### Trial sequence

Each trial started with the presentation of a fixation cross at the center of the screen for 500 ms. Then, a search display was presented until observers made a speeded response by pressing one of two mouse buttons (with the left- and right-hand index finger, respectively). Observers were instructed to search for the rotated ‘T’ and decide as quickly and accurately as possible whether the stem of the T was pointing to the left or the right. In case of a response error, a minus sign appeared on the screen for 1000 ms. An inter-stimulus interval of 1000 ms separated one trial from the next.

#### Design and procedure

Experiment 1 used a repeated-measures design, with the (within-subject) factors Context (old, new) and Epoch (1–8 (1–10), for Experiment 1A (1B), respectively). A set of 12 old-context displays with invariant arrangements of nontarget items was generated for each observer and repeated throughout the experiment. For new contexts, the configurations of nontarget items were generated randomly on each respective trial. Each old and new context was paired with two target locations (presented in different phases of the experiment). In order to rule out location probability effects, different sets of target locations were selected randomly for old and new contexts, such that, overall, 48 possible target locations were assigned to the displays. The orientation of the targets was random on each trial, whereas nontarget orientations were constant in old contexts. The second factor Epoch divided the experiment into equally sized consecutive bins (each bin consisted of 120 trials).

The experiments started with a practice block of 24 randomly generated displays to familiarize observers with the task. All subsequent experimental blocks consisted of 24 trials, 12 with old- and 12 with new-context displays presented in random order.

An example sequence of the three experimental phases in Experiment 1A is presented in Figure 1 (top half). Displays were presented with initial target locations (Target Location 1) in the first 15 blocks (aggregated into 3 epochs; learning phase). In 20 subsequent blocks (epochs 4–7) displays were presented with the second target locations (Target Location 2; relocation phase), followed by another 5 blocks (epoch 8) presenting displays again with initial target locations (return phase). Each of the two target locations was presented 20 times. After each block, observers took a short break and continued with the experiment at their own pace. Overall, observers completed 984 trials.

In Experiment 1B, the relocation phase was extended to 35 blocks (epochs 4–10; in total 1224 trials) and the return phase was removed. Note that relocated targets were presented more than twice as often as initial target locations.

#### Recognition test

After the search task observers were asked to perform a final recognition test. Observers completed 24 trials, in which they had to decide via mouse button responses whether a particular display had been shown previously (old) or not (new). All displays were presented with initial target locations because the explicit recognition of a given old context – if present at all – should be stronger for reliably learned context-target relations (see preconditions above). The response was non-speeded and no error feedback was provided.

### Results Experiment 1A

#### Search task

Individual mean error rates were calculated for each variable combination. Overall, observers made relatively few errors (2.1%), and a repeated-measures analysis of variance (ANOVA) with context (old, new) and epoch (1–8) as within-subject factors did not yield any significant main or interaction effects (all ps >.20).

Next, individual mean RTs were calculated for old and new contexts separately for each epoch and observer. Error trials and RTs exceeding an observer’s mean RT by ±2.5 standard deviations were excluded from the analyses. This outlier criterion led to the removal of 2.5% of all trials; the same procedure was applied in all subsequent experiments resulting in comparable exclusion rates. Greenhouse-Geisser corrected values are reported in case Mauchley’s test of sphericity was significant (*p*<.05).

**Figure 2:**
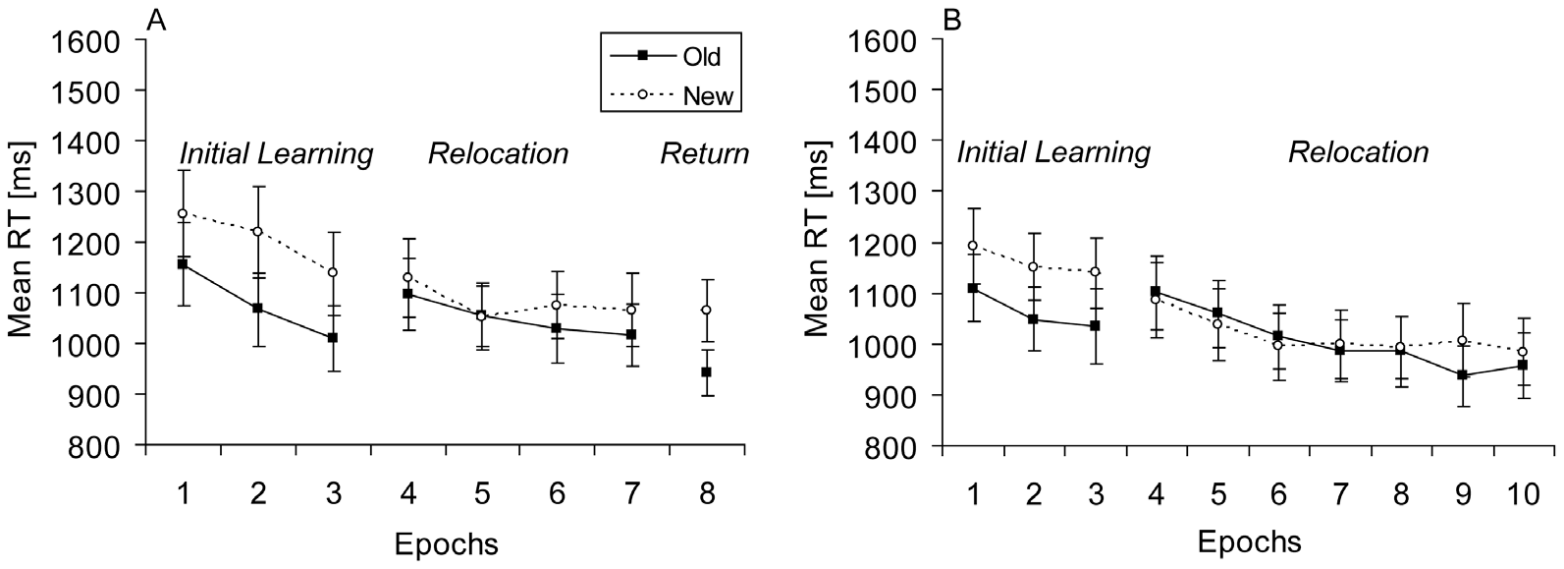
Results Experiment 1A and Experiment 1B. Mean RTs (in ms, and associated standard error bars) for old and new contexts (solid and dashed lines, respectively) as a function of epoch for (**A**) Experiment 1A and (**B**) Experiment 1B.

In a first step, individual mean RTs were computed for old and new contexts in each phase (learning, relocation, return). An overall ANOVA with the factors Context (old, new) and Phase (learning, relocation, return) was performed to examine whether contextual cueing changed in the different phases of the experiment. This analysis revealed significant main effects of context, *F*(1, 11) = 18.63, *p*<.01, and of phase, *F*(2, 22) = 16.01, *p*<.001, and a significant interaction between context and phase, *F*(2, 22) = 4.55, *p*<.05. Thus, contextual cueing was affected by the experimental phases (see Figure 2A). In order to explore the interaction effect, phases were analysed separately.

For the learning phase, an ANOVA with the factors Context (old, new) and Epoch (1–3) yielded significant main effects of context, *F*(1, 11) = 16.13, *p*<.01, and of epoch, *F*(2, 22) = 25.87, *p*<.001. RTs were on average 127 ms faster for old contexts as compared to new contexts and decreased by 132 ms across epochs. The interaction did not reach significance (*p*>.40), indicating that observers already showed a robust contextual-cueing effect in epoch 1. In order to determine in which block of the initial learning phase significant contextual cueing occurred first, RTs for old and new contexts were compared for each block. The first significant difference emerged in block 4, *t*(11) = 2.5, *p*<.05, which is comparable to findings of fast contextual learning in previous studies [28].

For the relocation phase, an ANOVA with the factors Context (old, new) and Epoch (4–7) revealed only a significant main effect of epoch, *F*(3, 33) = 8.29, *p*<.001, due to faster RTs (by 73 ms) in epoch 7 than in epoch 4. More important, there were no significant effects involving context (*ps*>.20), showing that there was no systematic contextual-cueing effect in the relocation phase.

Finally, in the return phase (epoch 8), the RT-difference between old and new contexts was again significant, *t*(11) = 4.37, *p*<.001. Additional comparisons based on blocks demonstrated that this difference was instantaneously significant in the first block of the return phase (i.e., in block 36), *t*(11) = 3.23, *p*<.01. In terms of magnitude, contextual cueing in the return phase was comparable to contextual cueing in the initial learning phase (124 vs. 127 ms, respectively).

#### Recognition test

Overall, the mean accuracy of recognizing old and new contexts was 59%. Observers correctly identified old contexts on 57% of trials (hit rate). The rate of reporting new contexts as old (false alarms) was significantly smaller (38.9%) than the hit rate, t(11) = 3.28, p<.01. To analyze whether observers’ ability to explicitly recognize old contexts was related to the size of contextual cueing, the individual sensitivity measure d′ (z(hits) - z(false alarms)) was computed and correlated with the contextual-cueing effects of the first and the second target locations. Observers’ ability to explicitly recognize old contexts was not significantly correlated with the mean contextual-cueing effects of neither the first nor the second target locations, r = -.17, p = .61 and r = .06, p = .86, respectively. This suggests that the explicit recognition of some of the displays [6] was not related to the occurrence of contextual cueing [29], [30].

### Discussion Experiment 1A

While contextual cueing occurred for initial target locations in the learning phase, visual search for relocated targets was only comparable to search in new-context displays in the relocation phase [12]–[14]. This suggests that old contextual associations were not adapted to the relocated targets. Furthermore, contextual cueing for initial target locations was preserved across the presentation of relocated targets [15], [16] and immediately facilitated search upon the return of initial target locations.

In Experiment 1B, the relocation phase was further prolonged to provide observers with an even larger number of repetitions of relocated targets to enable relearning to develop. The longer relocation phase was implemented because previous studies reported that successive implicit learning benefits from intensive training [17], [18].

### Results Experiment 1B

#### Search task

Relatively few errors occurred (2.4%) in Experiment 1B. A repeated-measures ANOVA with the factors Context (old, new) and Epoch (1–10) did not yield any significant main or interaction effects (all ps >.30).

First, to analyze RTs, an overall ANOVA with the factors Context (old, new) and Phase (learning, relocation) revealed significant main effects of context, *F*(1, 11) = 30.25, *p*<.001, and of phase, *F*(1, 11) = 19.72 *p*<.01), as well as a significant interaction between context and phase, *F*(1, 11) = 6.22, *p*<.01. Because the phases affected contextual cueing (see Figure 2B) separate analyses follow.

For the learning phase, a context (old, new) by epoch (1–3) ANOVA yielded a significant main effect of context, *F*(1, 11) = 17.33, *p*<.01, and a typical main effect of epoch, *F*(2, 22) = 5.67, *p*<.05. RTs were on average 97 ms faster for old contexts in comparison to new contexts. The interaction between context and epoch was not significant (*p*>.70). Additional analyses based on blocks revealed that the first significant difference in RTs between old and new contexts emerged in block 5, *t*(11) = 3.34, *p*<.01.

Next, for the relocation phase, an ANOVA with the factors Context (old, new) and Epoch (4–10) revealed a significant main effect of epoch, *F*(6, 66) = 17.56, *p*<.001, and a significant interaction between context and epoch, *F*(6, 66) = 3.09, *p*<.05. Subsequent comparisons between RTs for old and new contexts performed separately for each epoch revealed a significant difference only in epoch 9 (68 ms), *t*(11) = 2.73, *p*<.05, but not in any other epoch of the relocation phase (mean contextual cueing = 8 ms). The significant contextual-cueing effect in epoch 9 appears to be an isolated outlier effect rather than a systematic contextual-cueing effect.

#### Recognition test

Overall, the mean accuracy in the recognition test was 48.6%. Observers’ hit rate of 57.6% was comparable to the false alarm rate of 60.4%, t(11) = .60, p>.50, suggesting that observers were mostly unaware of the display repetitions [4].

### Discussion

Experiment 1 investigated relearning of contextual associations after a change of the target locations with different presentation times. Observers showed robust contextual cueing in the initial learning phases. However, after target relocation, contextual cueing was greatly reduced and remained insignificant across the shorter and longer relocation phases [12]–[14]. Overall, the results of Experiment 1 suggest that an increased amount of training is not sufficient to enable relearning of relocated targets in contextual cueing.

In the final return phase, initial target locations elicited reliable contextual-cueing effects, which were comparable to cueing-effects in the learning phase. This result suggests that contextual memory for the initially learned target locations was stable and unaffected by the repeated presentation of the same displays with relocated targets, which is in line with previous findings of enduring contextual cueing [10], [15], [16], [31].

## Experiment 2

Experiment 2 examined contextual new-learning under identical training conditions as used for relearning in Experiment 1. New-learning also involved a critical change after an initial learning phase: a new contextual cueing experiment started presenting an entirely new set of old- and new-context displays (see Figure 1, bottom half). Like Experiment 1A, Experiment 2A had an initial learning phase (3 epochs), followed by a new-learning phase (4 epochs) and a final return phase (1 epoch). Like Experiment 1B, Experiment 2B comprised of an extended new-learning phase of seven epochs and no return phase.

### Method

Apparatus, stimuli, design, and procedure were similar to Experiment 1, except that in Experiment 2, observers were presented with one set of 12 old-context displays (old contexts 1–12) in the learning phase (epochs 1–3) and a further distinct set of 12 old-context displays (old contexts 13–24) in subsequent epochs of the new-learning phase.

Figure 1 (bottom half) illustrates the sequence of experimental phases for Experiment 2A. Subsequent to learning (epochs 1–3), further old-context displays were presented from epoch 4 to epoch 7 (new-learning phase), which was followed by the presentation of old-context displays from the initial learning phase in epoch 8 (return phase). In Experiment 2B, epochs 4–10 represented the new-learning phase, which was not followed by a return phase.

In total, 24 old-context displays with 24 different target locations (12 for each set of displays) were generated for each observer. Another 24 different target locations were assigned to new-context displays. In the final recognition test, observers completed 48 trials including the 24 old-context displays and 24 randomly generated displays.

Twelve adults (9 women) took part in Experiment 2A with a mean age of 27.8 years (range: 22–49 years). Another 12 adults (11 women) were tested in Experiment 2B with a mean age of 26.6 years (range: 21–32 years). All observers reported normal or corrected-to-normal visual acuity and were right-handed. They received either payment (8 or 10 €) or course credits for their participation.

### Results Experiment 2A

#### Search task

Overall, observers made few errors (2.8%). A repeated-measures ANOVA with the factors Context (old, new) and Epoch (1–8) revealed no significant effects (all ps >.20).

**Figure 3:**
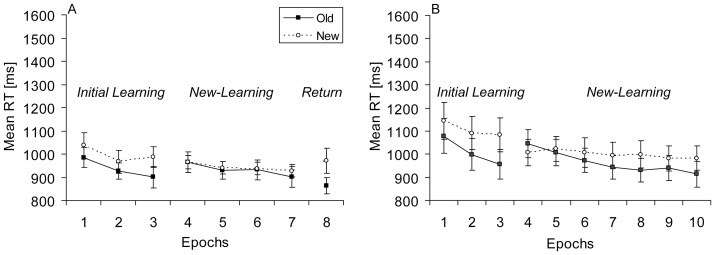
Results Experiment 2A and Experiment 2B. Mean RTs (in ms, and associated standard error bars) for old and new contexts (solid and dashed lines, respectively) as a function of epoch for (**A**) Experiment 2A and (**B**) Experiment 2B.

For the analysis of RTs, an overall ANOVA with the factors Context (old, new) and Phase (learning, new-learning, return) yielded significant main effects of context, *F*(1, 11) = 13.09, *p*<.01, and of phase, *F*(2, 22) = 6.32, *p*<.01, as well as a significant interaction between context and phase, *F*(1.17, 12.85) = 10.48, *p*<.01. Because the interaction indicates that contextual cueing was affected by the experimental phases (see Figure 3A), separate analyses were performed.

For the learning phase, a context (old, new) by epoch (1–3) ANOVA yielded significant main effects of context, *F*(1, 11) = 19.43, *p*<.01, and of epoch, *F*(2, 22) = 8.05, *p*<.01. RTs were on average 78 ms faster for old contexts than for new contexts. The interaction between context and epoch was not significant (*p*>.70). Additional analyses based on blocks showed that RTs of old and new contexts started to differ significantly in block 2, *t*(11) = 2.44, *p*<.05.

For the new-learning phase, a context (old, new) by epoch (4–7) ANOVA revealed only a main effect of epoch, *F*(3, 33) = 4.84, *p*<.01. The main effect of context and the interaction between context and epoch were not significant (*ps*>.10), and mean contextual cueing was −5 ms.

Finally, in the return phase (epoch 8), RTs were again faster (by 146 ms) for old contexts than for new contexts, *t*(11) = 4.62, *p*<.001, and this difference already emerged in the second block of the return phase (i.e., block 37), *t*(11) = 3.36, *p*<.01.

#### Recognition test

Overall, mean accuracy in the recognition test was 47.2%. Observers showed a hit rate of 51.4% and a false alarm rate of 52.1% for the first set of displays, t(11) = .16, p>80. A similar pattern of hits (48.6%) and false alarms (59%) was observed for the second set of displays, t(11) = 1.7, p>.10, suggesting that neither the first nor the second set of old-context displays was recognized explicitly.

### Discussion Experiment 2A

Successful new-learning was not observed after four epochs of training in Experiment 2A [17]. At the same time, the presentation of further old-context displays did not affect contextual cueing for initially learned contexts in the return phase [15], [16]. Similar to Experiment 1B, new-learning was tested with an even longer training phase in Experiment 2B [17].

### Results Experiment 2B

#### Search task

In Experiment 2B, few errors occurred (3.1%). A repeated-measures ANOVA with the factors Context (old, new) and Epoch (1–10) revealed no significant effects (all ps >.40).

An overall ANOVA on the mean RTs with the factors Context (old, new) and Phase (learning, new-learning) yielded significant main effects of context, *F*(1, 11) = 9.85, *p*<.01, and of phase, *F*(2, 22) = 6.08, *p*<.05. The interaction between context and phase was only marginally significant, *F*(1, 11) = 4.92, *p* = .05, suggesting that the effect of phase on contextual cueing was now reduced (see Figure 3B).

For the learning phase, a context (old, new) by epoch (1–3) ANOVA yielded significant main effects of context, *F*(1, 11) = 16.34, *p*<.01, and of epoch, *F*(2, 22) = 11.85, *p*<.001. RTs were on average 96 ms faster for old contexts than for new contexts. The interaction between context and epoch reached marginal significance, *F*(2, 22) = 3.04, *p* = .07, suggesting that the RT-difference between old and new contexts increased from epoch one (67 ms) to epoch three (127 ms). Further analyses based on blocks showed that the first significant difference between old and new contexts manifested in block 3, *t*(11) = 3.02, *p*<.05.

For the new-learning phase, a context (old, new) by epoch (4–10) ANOVA revealed a significant main effect of epoch, *F*(3.03, 33.29) = 6.34, *p*<.01, and no main effect of context (*p*>.20). In addition, the interaction between context and epoch was significant, *F*(6, 66) = 4.72, *p*<.01, reflecting a gradual increase of contextual cueing across epochs. An additional ANOVA with the factors Context (old, new) and Epoch (7–10) revealed a marginally significant main effect of context, *F*(1, 11) = 4.36, *p* = .06, representing a sustained mean cueing effect of 57 ms across epochs 7 to 10.

#### Recognition test

Overall, the mean accuracy in the recognition test was 47.4%. The hit rate for the first set of displays was 45.8%, which was comparable to the false alarm rate of 56.9%, *t*(11) = 1.36, *p*>.10. A similar pattern of hits (55.6%) and false alarms (54.9%) was observed for the second set of displays, *t*(11) = .096, *p*>.90. Overall, this pattern of results suggests that observers were not able to recognize the old-context displays.

### Discussion

Experiment 2 was designed to investigate successive learning of new contextual information based on intensive training. Contextual cueing was observed for a first set of old-context displays in the initial learning phases, but not for a second set of old-context displays in the shorter version of the new-learning phase. However, more intensive training facilitated the development of contextual new-learning (at least to some extent) – a finding that was not observed for relocated targets inserted into old contexts in the longer version of the training phase in Experiment 1B. As in Experiment 1 of the present study, old-context displays from the initial learning phase elicited large contextual-cueing effects in the return phase.

In sum, the results of Experiment 2 indicate that the acquisition of new context-target associations might develop gradually in contextual cueing. Although the contextual-cueing effect in the longer version of the new-learning phase was relatively small, the results nevertheless indicate that contextual new-learning may gradually increase with training. This finding is in agreement with results presented by [17], who showed that successive learning of two statistical regularities was only observed when the exposure with the second regularity was tripled in time in comparison to the exposure with the first regularity. However, the learning effect was still significantly smaller for the second regularity than for the first regularity – mirroring the current results in Experiment 2B.

By contrast, a similar trend was not observed for adaptation to relocated targets in Experiment 1B, which suggests that old contextual associations might be limited to a single target location [10]. Two further experiments explored this difference between relearning and new-learning by introducing an overnight break between subsequent learning phases.

## Experiment 3

Experiment 3 tested adaptation to relocated targets after a 24-hour break. The experimental design was similar to Experiment 1A, except that the phases of the experiment were performed on two consecutive days. Observers completed the initial learning phase (3 epochs) on one day, and the relocation (7 epochs) and return phases (1 epoch) on the next day. The 24-hour break was introduced because successive contextual learning is facilitated by sleep breaks between learning sessions [15], [16]. Therefore, our manipulation may also enhance adaptation to relocated targets.

### Method

Apparatus, stimuli, design, and procedure were similar to Experiment 1A, except that observers completed a total of 1368 trials on two consecutive days. As in Experiment 1A, each old- and new-context display was paired with two different target locations, of which the first target location was presented on the first day for 3 epochs (initial learning phase). On the next day, the same old-context displays were presented with the second target locations for 7 epochs (relocation phase), immediately followed by the presentation of initial target locations in epoch 11 (return phase; see top half of Figure 1). The experiment started with a practice block on both days. The recognition test was administered on the second day. Experimental sessions were separated by approximately 24 hours.

Fourteen adults (12 women) took part in the experiment with a mean age of 26.8 years (range: 19–45 years). All observers reported normal or corrected-to-normal visual acuity; one observer was left-handed. Observers received either payment (14€) or course credits.

### Results

#### Search task

Overall, observers made few errors (2.1%), and an ANOVA with the factors Context (old, new) and Epoch (1–11) did not result in any significant effects (all ps >.10).

**Figure 4:**
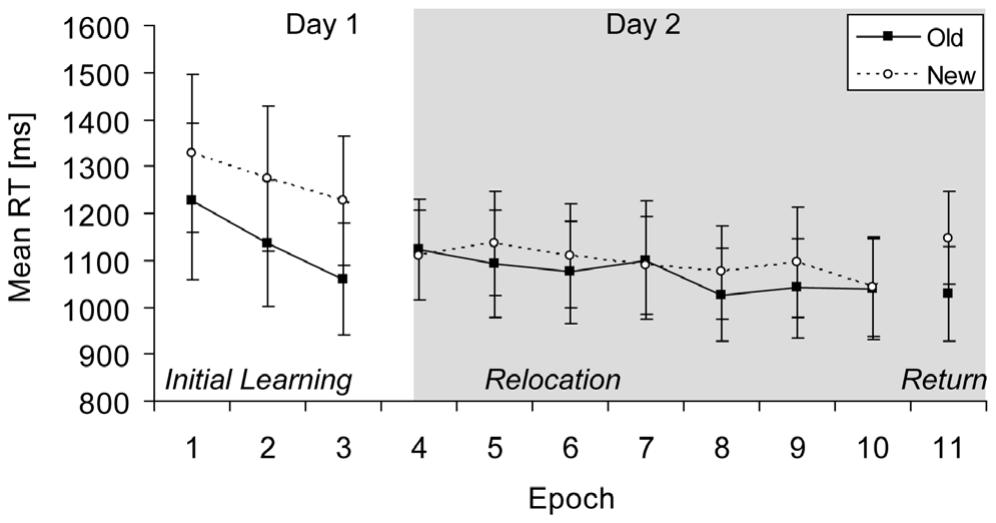
Results Experiment 3. Mean RTs (in ms, and associated standard error bars) for old and new contexts (solid and dashed lines, respectively) as a function of epoch.

For the analysis of RTs, first, an overall ANOVA with the factors Context (old, new) and Phase (learning, relocation, return) was performed, which revealed significant main effects of context, *F*(1, 13) = 13.69, *p*<.01, and of phase, *F*(1.20, 15.55) = 5.65, *p*<.05, and a significant interaction between context and phase, *F*(2, 26) = 5.34, *p*<.05. Since the factor Phase affected contextual cueing (see Figure 4) separate analyses follow.

For the learning phase, an ANOVA with the factors Context (old, new) and Epoch (1–3) yielded significant main effects of context, *F*(1, 13) = 15.45, *p*<.01, and of epoch, *F*(1.14, 14.84) = 8.99, *p*<.01. RTs were on average 135 ms faster for old in comparison to new contexts. The interaction between context and epoch was marginally significant, *F*(2, 26) = 3.14, *p* = .06, reflecting an increase in the contextual-cueing effect from epoch 1 (102 ms) to epoch 3 (166 ms). Subsequent RT comparisons of old and new contexts in each block revealed the first significant difference in block 4, *t*(13) = 2.57, *p*<.05.

For the relocation phase performed on the next day, an ANOVA with the factors Context (old, new) and Epoch (4–10) revealed a significant main effect of epoch, *F*(2.52, 32.77) = 6.39, *p*<.01. The main effect of context and the interaction between context and epoch were not significant (*ps*>.10). Mean contextual cueing was 23 ms.

Finally, in the return phase, when the initial target locations returned, RTs for old contexts were 118 ms faster than for new contexts, *t*(13) = 3.01, *p*<.05, and this difference occurred again instantaneously in the first block of the return phase (i.e., block 51), *t*(13) = 2.54, *p*<.05.

#### Relearning across experiments

A further analysis was computed to examine whether the length of the relocation phases facilitated relearning. To this end, mean contextual cueing was computed for the basic relocation phase in Experiment 1A (epochs 4–7) and compared to mean contextual cueing of the extension of the relocation phase (epochs 8–10) in Experiments 1B and 3. Data of Experiments 1B and 3 were collapsed for this analysis, as there was no difference in contextual cueing between the respective extensions (epochs 8–10) of the relocation phases, t(24) = 0.02, p>.40 (one-tailed). An independent t-test revealed no significant difference between mean contextual cueing of basic and extended training, t(36) = .097, p>.40 (one-tailed). This means, basic and extended training resulted in similarly insignificant contextual-cueing effects for relocated targets (32 ms vs. 34 ms, respectively).

#### Distance analysis

**Figure 5:**
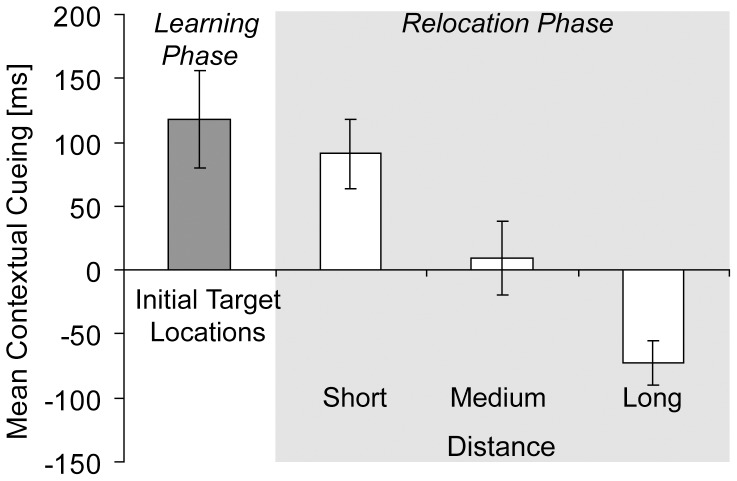
Distance analysis. Mean contextual cueing (in ms, and associated standard error bars) for initial target locations (collapsed across the learning phases of all relocation experiments; gray bars), and mean contextual cueing (in ms, and associated standard error bars) for relocated targets (white bars) separately for short, medium and long distances relative to the initial target locations.

An additional analysis was computed on the collapsed data (n = 38) of all relocation experiments (Experiments 1A, 1B, & 3) to examine whether relocated targets elicited contextual cueing when they were located in proximity or farther away from initially learned target locations. Mean distances between initial target locations and relocated targets (range: 2.5°–19.5°) were separated into three equal groups, resulting in a “short distance” (M = 4.9°), a “medium distance” (M = 9.1°) and a “long distance” group (M = 14.4°). Figure 5 depicts contextual cueing of relocated targets in each distance group and in relation to overall contextual cueing of initial target locations. A one-way ANOVA was performed on contextual cueing of relocated targets with the factor Distance (short, medium, long), which revealed a significant effect of distance on contextual cueing of relocated targets, F(2, 111) = 6.74, p<.01. Contextual cueing of relocated targets at short distances was comparable to contextual cueing of initial target locations (p>.60). By contrast, contextual cueing of relocated targets at medium and long distances was significantly smaller than contextual cueing of initial target locations (ps <.01); relocated targets at long distances even elicited contextual costs [10]; [14].

#### Recognition test

Overall, the mean accuracy in the recognition test was 55.4%. The difference between hits (54.2%) and false alarms (43.5%) was marginally significant, t(13) = 2.15, p = .05. As in Experiment 1A, the individual sensitivity measure d′ (z(hits) - z(false alarms)) was computed as a measure of explicit recognition performance and correlated with the contextual-cueing effects for the first and the second target location. Observers’ ability to explicitly recognize old contexts was not significantly correlated with the mean contextual-cueing effects of neither the first nor the second target location, r = .42, p>.10 and r = −.16, p>.50, respectively. Thus, the ability to explicitly recognize some of the displays was not related to contextual cueing [29], [30].

### Discussion

In Experiment 3 observers completed two learning sessions on two consecutive days to investigate relearning of existing contextual associations. Robust contextual cueing was observed in the initial learning phase on the first day. On the second day, contextual cueing did not occur for relocated targets, although observers were trained with numerous repetitions. Contextual cueing for relocated targets was, in fact, similar to the insignificant effects observed in Experiment 1. In addition, contextual cueing during the extension of the relocation phase (relative to shorter training in Experiment 1A) was comparable in Experiments 1B and 3. Hence, the additional overnight break implemented in Experiment 3 did not facilitate contextual relearning, although sleep is known to reduce proactive interference in some cases [15].

However, an additional analysis performed on the collapsed data of all relearning experiments revealed that repositioned targets located in the proximity of initial target positions did elicit contextual-cueing effects. This interesting finding suggests that contextual cues guided visual search to initially learned target regions. Hence, relocated targets presented inside these regions were detected faster than relocated targets positioned further away [10], [14]. With increasing distances, substantial contextual costs emerged, suggesting that attention was in fact “misguided” after target relocation. Because attention was continuously guided to initial target locations, contextual cueing was immediately observed in the return phases of Experiments 1A and 3.

Overall, the observed lack of genuine adaptation to relocated targets in Experiment 3 suggests that the conditions known to facilitate contextual new-learning [15], [16] do not increase the likelihood for relearning to occur. Rather, relearning of previously learned contextual associations appears to be fairly restrained.

## Experiment 4

Experiment 4 was conducted to investigate whether new-learning occurs after a 24-hour break [15], [16]. The experiment was identical to Experiment 2A, except that a further set of old-context displays was presented during the new-learning phase on the second day of the experiment (see Experiment 2).

### Method

Apparatus, stimuli, design, and procedure were similar to Experiment 3, except that a further set of old-context displays was presented during a new-learning phase on the second day (see bottom half of Figure 1, and Experiment 2A). The final recognition test required observers to complete 48 trials including the 24 old-context displays and 24 randomly generated novel displays.

Fourteen adults (13 women) took part in the experiment with a mean age of 22.4 years (range: 18–30 years). All observers reported normal or corrected-to-normal visual acuity; one observer was left-handed. Observers received either payment (14€) or course credits.

### Results

#### Search task

Overall, few errors occurred (1.9%). An ANOVA with the factors Context (old, new) and Epoch (1–11) revealed a significant main effect of context, F(1,13) = 8.65, p<.05, reflecting fewer errors for old contexts (1.7%) than for new contexts (2.2%).

**Figure 6:**
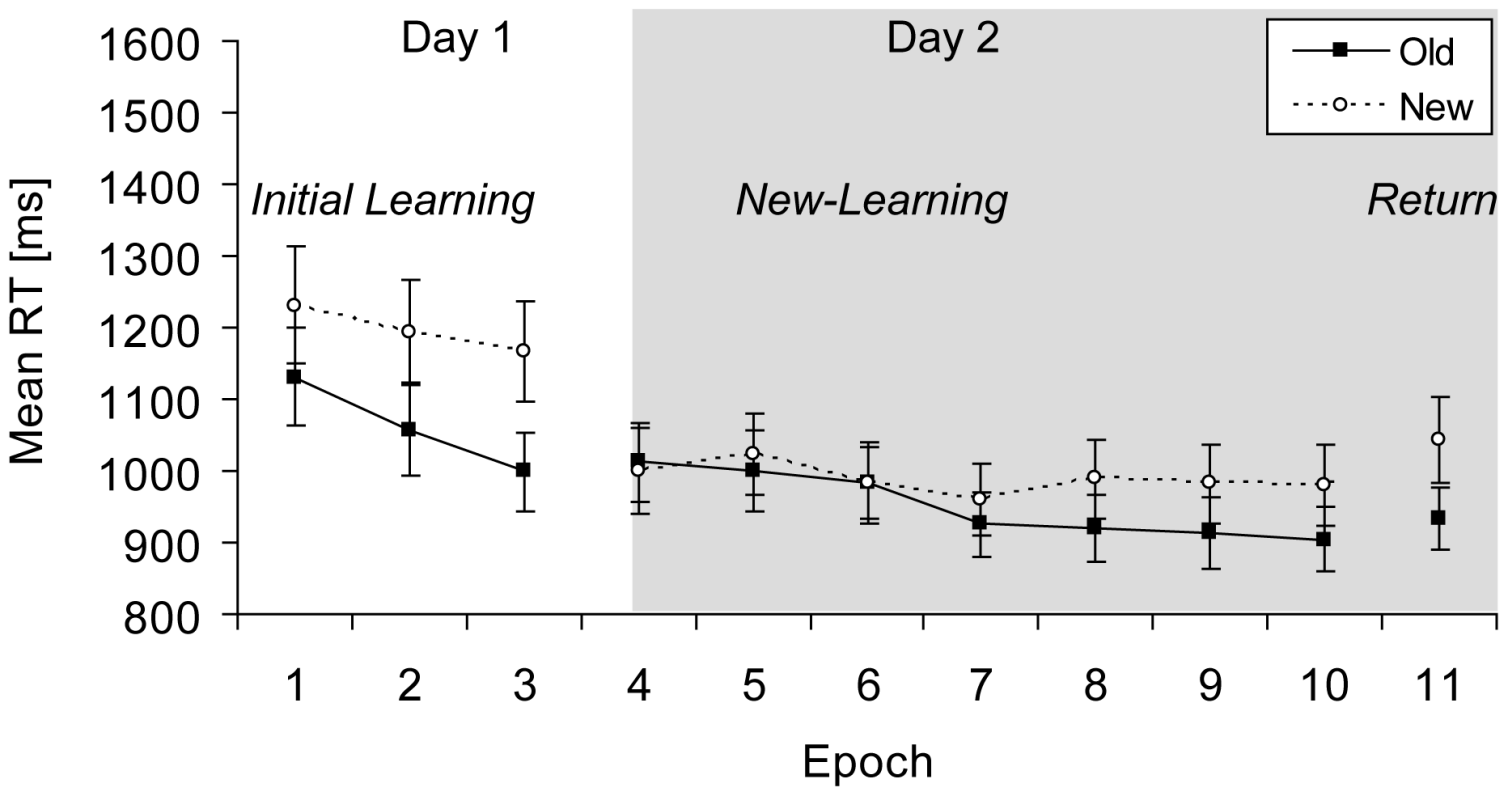
Results Experiment 4. Mean RTs (in ms, and associated standard error bars) for old and new contexts (solid and dashed lines, respectively) as a function of epoch.

Mean RTs for old and new contexts across epochs are depicted in Figure 6. An overall ANOVA on the RT data with the factors Context (old, new) and Phase (learning, new-learning, return) yielded significant main effects of context, *F*(1, 13) = 14.33, *p*<.01, and of phase, *F*(2, 26) = 20.37, *p*<.001. The interaction between context and phase was also significant, *F*(2, 26) = 8.15, *p*<.01. Separate analyses follow.

For the learning phase, an ANOVA with the factors Context (old, new) and Epoch (1–3) yielded main effects of context, *F*(1, 13) = 18.34, *p*<.01, and of epoch, *F*(2, 26) = 9.57, *p*<.01. RTs were on average 135 ms faster for old relative to new contexts. The interaction between context and epoch was not significant (*p*>.10). An additional analysis based on blocks revealed that the first (marginally) significant difference in RTs between old and new contexts occurred in block 2, *t*(13) = 2.10, *p* = .06.

Next, for the new-learning phase performed on the second day, an ANOVA with the factors Context (old, new) and Epoch (4–10) revealed a significant main effect of epoch, *F*(6, 78) = 10.15, *p*<.001. The main effect of context was not significant (*p*>.10), but the interaction between context and epoch was significant, *F*(6, 78) = 4.44, *p*<.01, reflecting an increase in contextual cueing across epochs. An additional ANOVA with the factors Context (old, new) and Epoch (7–10) revealed a significant effect of context for epochs 7 to 10, *F*(1, 13) = 6.91, *p*<.05, showing a sustained mean contextual benefit of 62 ms for epochs 7 to 10.

Finally, in the return phase (on the second day), when the first set of displays returned, RTs were on average 110 ms faster for old contexts than for new contexts, *t*(13) = 3.56, *p*<.001. Similar to all previous return phases of the present study, contextual cueing was observed early in the return phase (i.e., in block 51), *t*(13) = 2.33, *p*<.05.

#### New-learning across experiments

A further analysis was computed to examine whether the extended length of the new-learning phase facilitated new-learning. Mean contextual cueing was computed for the basic new-learning phase in Experiment 2A (epochs 4–7) and compared to the extension of the new-learning phase (epochs 8–10) in Experiments 2B and 4 (data collapsed as there was no significant difference in contextual cueing between Experiments 2B and 4, t(24) = .31, p>.30, one-tailed). An independent t-test revealed a significant difference in contextual cueing between basic and extended training, t(36) = 1.94, p<.05 (one-tailed), indicating that contextual cueing was larger after extended learning than after basic training (65 ms vs. −5 ms, respectively).

#### Relearning vs. new-learning

In a final step, we compared contextual-cueing effects between the collapsed data of Experiment 1B and 3 (relearning, n = 26) and Experiment 2B and 4 (new-learning, n = 26). Contextual-cueing effects were computed for epochs 8–10 and entered into a repeated-measures ANOVA with the within-subject factor Epoch (8–10) and the between-subject factor Experiment (relearning, new-learning). The interaction between epoch and experiment was significant, F(2, 100) = 3.58, p<.05, reflecting a larger contextual-cueing effect for new-learning (65 ms) compared to a smaller and more varying cueing effect for relearning (34 ms) across the last 3 epochs (main effect of epoch, F(2, 100) = .82, p>.40]. This outcome indicates that, with sufficient training, new-learning is more effective than relearning of established associations.

#### Recognition test

Overall, the mean accuracy in the recognition test was 47.5%. For the first set of displays, the number of hits (54.2%) was comparable to the rate of false alarms (52.5%), t(11) = .30, p>.70. A similar pattern of hits (54.8%) and false alarms (46.4%) was found for the second set of displays, t(11) = 1.16, p>.20. Therefore, observers did not explicitly recognize the old context-displays.

### Discussion

The results of Experiment 4 showed contextual learning for two sets of repeated displays when learning was performed on two consecutive days. Observers revealed a robust contextual-cueing effect for a first set of old-context displays in the initial learning phase. Subsequently, a contextual-cueing effect developed for a second set of old-context displays in the new-learning phase on the next day, which was larger than the effect observed after shorter training in Experiment 2A. Simultaneously, contextual new-learning in Experiment 4 was comparable to the results of Experiment 2B that implemented the same amount of training, but no break between phases. Reliable contextual cueing was also observed for the first set of old-context displays in the return phase.

This pattern of results shows that two sets of old-context displays can be learned on two consecutive days [16]. However, contextual new-learning did not develop as fast as initial learning on the first day. Previous studies [15], [16], [32] have already suggested that new-learning may not be as successful as initial learning in contextual cueing. Nevertheless, new-learning was reliable in Experiment 4, which means that our training conditions effectively facilitated new-learning. By contrast, relearning in Experiment 3 was clearly not observed under identical training conditions.

### Analysis of Excluded Observers

Additional analyses were performed to examine the development of learning for those observers who did not show (above zero) contextual-cueing effects in the initial learning phases (baseline contextual cueing), and who were therefore excluded from the main analyses (see Method, Experiment 1). Data of excluded observers in the relearning experiments were collapsed (4, 10, & 7 observers in Experiment 1A, 1B, & 3, respectively ), and mean RTs for old and new contexts were computed for each phase (initial learning, relocation, return) and entered into paired-sample *t* tests. The same procedure was used for excluded observers of new-learning experiments (initial learning, new-learning, return; 4, 9, & 4 observers in Experiment 2A, 2B, & 4, respectively).

**Figure 7:**
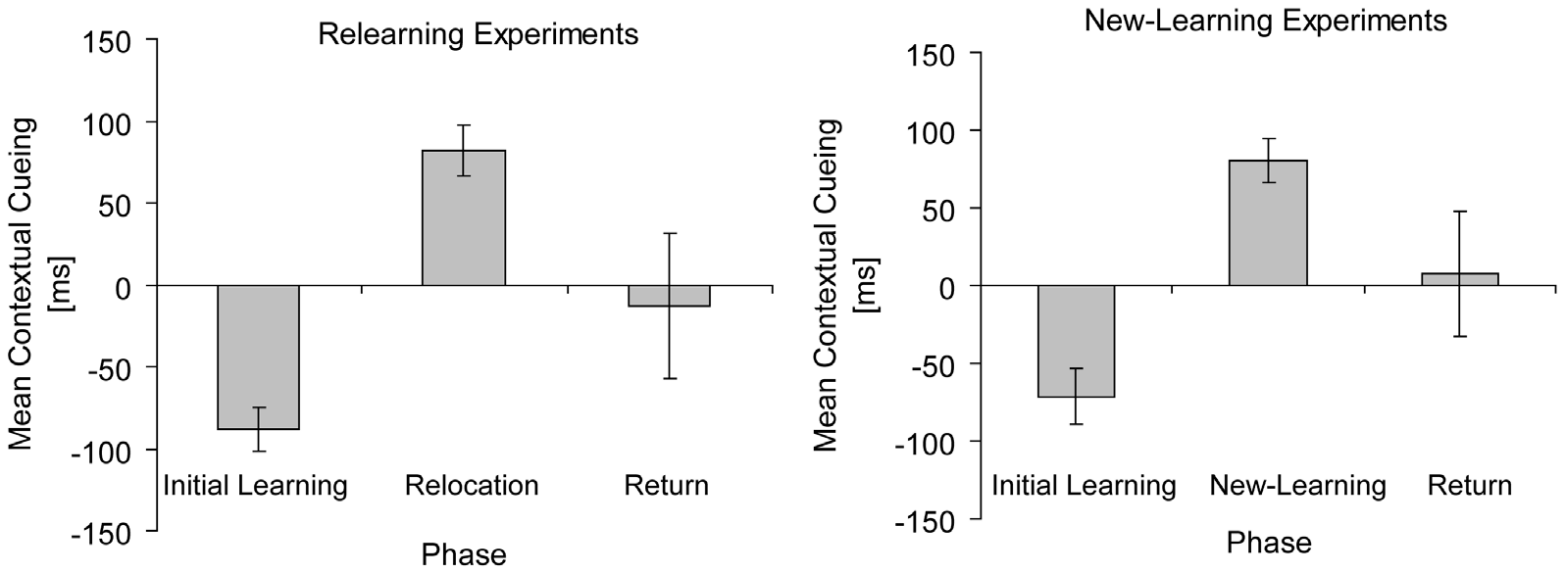
Results of excluded observers. Mean contextual cueing (in ms, and associated standard error bars) of excluded observers for relearning experiments (left panel) and the respective experimental phases (initial learning, relocation, return) and for new-learning experiments (right panel) and the respective experimental phases (initial learning, new-learning, return).

Figure 7 presents mean contextual cueing effects of excluded observers for each phase of the relearning (left panel) and new-learning experiments (right panel). In the relearning experiments (Experiment 1A, 1B, & 3), excluded observers’ (n = 21) search RTs were on average 88 ms slower for old compared to new contexts in the initial learning phases, *t*(21) = 6.54, *p*<.01. Subsequently, however, significant contextual cueing (82 ms) was observed for relocated targets in the relocation phases, *t*(20) = 5.29, *p*<.01. Contextual cueing for initial target locations (−13 ms) was not significant in the return phases of the two respective experiments (Experiments 1A & 3), *t*(10) = .29, *p*>.70.

Similarly, the analysis of the excluded observers (n = 17) in the new-learning experiments (Experiments 2A, 2B, & 4) showed negative contextual cueing (−71 ms) in the initial learning phases, *t*(16) = 3.96, *p*<.01, and no contextual cueing for initial old contexts in the final return phases (7 ms), *t*(7) = .18, *p*>.80 (Experiments 2A & 4). However, contextual cueing was significant in the new-learning phases (81 ms) following initial (unsuccessful) learning, *t*(16) = 5.53, *p*<.01.

Thus, even though a group of observers failed to learn old contexts in the initial learning phases of the six reported experiments, they showed contextual cueing in the subsequent relocation and new-learning phases. Thus, a number of observers can be characterized as “late” learners of repeated spatial contexts. The results of these late learners in the relearning experiments imply that contextual cueing is limited to a single target region for a given old context, consistent with the main findings of the present study.

Luhmann (2011, [22]) also presented two target locations sequentially and observed more contextual cueing in the relocation phase compared to the initial learning phase. Several explanations could account for the difference between early and late learning: On the one hand, late learners possibly required additional time to adopt a “passive”, rather unfocused mode of search known to facilitate contextual learning [33]. On the other hand, because contextual cueing lacks test-retest reliability [16] early and late learning could be coincidental for each individual. Also, early learners might have encountered easy-to-learn displays in the initial learning phase, agreeing with the observation that some old contexts are learned more easily than others ([34]; we would like to thank an anonymous reviewer for this suggestion). In sum, the ability to learn repeated contexts probably exists in every normal adult, but whether learning is actually revealed in a test session may depend on a variety of factors.

## General Discussion

The aim of the present study was to compare and contrast memory adaptation in relearning of existing contextual associations with successive learning of new contextual associations. To this end, we examined contextual relearning and contextual new-learning under identical training conditions. During relearning, target items were relocated to a previously empty display location within their respective invariant context. Relearning was observed neither after intensive training (Experiment 1) nor after an extended (24-hour) break including sleep (Experiment 3).

Contextual new-learning was examined with the successive presentation of two distinct sets of invariant contexts realized under the same training conditions as used for relearning. The results showed that new-learning did not benefit from relatively short training (Experiment 2A); but when the training phase was further prolonged, a contextual-cueing effect developed for a second set of invariant contexts (Experiment 2B). Similarly effective contextual new-learning was observed after an overnight break (Experiment 4).

Interestingly, robust contextual cueing for initially learned contexts was found in all return phases, irrespective of successful (Experiment 4) or unsuccessful learning in the meantime (Experiments 1A, 2A, 3). Reliable contextual-cueing effects were observed immediately once the initially learned contexts returned. This indicates that established associations for old contextual layouts presented in the initial learning phases were readily available after extended interludes. In sum, initially learned contexts were not affected by either relocated targets or by further (newly introduced) old-context displays.

Most of the previous studies that examined adaptation to relocated targets focused on the immediate consequences of target relocations on contextual cueing, and failed to observe cueing effects for relocated targets [12]–[14]. Even when relocated targets elicited contextual cueing, a cueing effect was not observed for initial target locations [22]. In Luhmann’s (2011, [22]) study, each of two target locations was presented for five repetitions in sequential order, and contextual cueing was observed only for the second target location, but not the first location – an outcome that is comparable to the results of the late learners in the present study. Here, we greatly increased the number of presentations of relocated targets and introduced a break, but adaptation to relocated targets was still not reliably obtained. While no contextual cueing occurred after target relocation, there was also no overall contextual cost, replicating previous studies that reported no or only transient costs directly after target relocation [12]–[14]. The overall lack of contextual costs suggests that search did not continue to be guided to initial target locations. However, targets relocated to positions in the proximity of initial target locations still benefited from contextual cueing, whereas relocated targets positioned further away suffered substantial contextual costs. This pattern indicates that visual search was in fact continuously guided to (the region around) the initial target locations [10], [14]. Continuous guidance to initial target locations by old contexts may also explain why contextual cueing readily occurred in the return phases of the relearning experiments. Overall, our results further support the view that relearning is restricted because contextual cueing is essentially limited to a single target location (or region) [10].

Unlike adaptation to relocated targets, new-learning developed after extended training. This suggests that training can facilitate contextual new-learning to some extent, in accordance with findings from other implicit-learning tasks [17], [18]. But unlike previous studies on contextual cueing [15], [16], successful new-learning was not substantially enhanced after sleep when the same amount of training was applied. In general, sleep should reduce proactive interference; that is, active old memories should interfere less with the acquisition of new memories after sleep (see [35], [36] for overviews regarding memory-based interference effects). If proactive interference did impair new-learning in the present study, its effect already subsided over the course of standard training, whereas sleep was not critical for (and did not add to) the reduction of proactive interference. Previous studies on contextual new-learning either only tested contextual new-learning after overnight breaks without implementing different training durations [16], or training phases were considerably shorter than in our study and did not follow initial learning immediately [15]. Thus, our investigation is, to the best of our knowledge, the first to show that long new-learning phases, which immediately follow initial learning, promote new-learning as efficiently without as much as with sleep breaks. In line with findings relating to other implicit learning-tasks [17], [18], [21], our results consequently support the view that the number of repeated exposures is the most influential contributor to successful implicit new-learning.

While new-learning of further, previously unseen contexts was observed in the present study, old contextual associations were not relearned to incorporate permanently relocated targets. Consequently, relearning contextual associations does not appear to be a “simple” case of learning novel contextual information – if this were the case, relearning should develop once proactive interference is reduced. Rather, in addition to proactive interference, further factors seem to impede adaptation to relocated targets. Specifically, supported by findings of [10], we propose that predictive contexts can only be associated with a single target location, constraining any further adaptive processes.

In contrast to our results, research using the incidental Serial Reaction Time (SRT) task suggests that relearning of implicit associations between predictors and targets was achieved with no difficulty [37]. In this study, observers were trained with a sequence of individually presented cues that predicted the (likely) continuation of that sequence. That is, cues were associated with more or less probable outcomes (similar to the pairing between spatial context and target location in contextual cueing). After training, the cue sequences were paired with new outcomes, and observers were able to successfully associate old cues with new outcomes. Hence, relearning of old associations was observed in an SRT-task, but not for contextual cueing in the present study.

Even though contextual cueing and SRT tasks (as well as other implicit learning tasks) share several similarities, the specifics of the tasks as well as the underlying learning mechanisms might nevertheless differ substantially (see [38] for review; see also [39]). In general, observers learn spatio-temporal sequences and rather simple associations between one cue and one highly probable outcome in SRT tasks, whereas contextual cueing involves the acquisition of more complex spatial associations between *multiple* (context-) objects and a definite target location [5]. These general differences could explain why adaptation occurs readily in SRT learning, but not in contextual (re-)learning. More specifically, in the study by [37] old cues were used as both cues and outcomes to realize new associations in the relearning phase. Hence, new outcomes were familiar objects, which could have facilitated relearning. Indeed, [13] have reported contextual cueing for two familiar target locations associated with the same invariant context [24]. In Experiment 2 of their study, search displays always contained two targets at two different locations (one was oriented left/right, one was pointing up−/downward). Observers searched for one of the targets in one half of the experiment and for the other target in the other half. Reliable contextual cueing was observed for both target locations, due to their simultaneous, and thus predictable, presentation. By contrast, target relocations were unpredictable and introduced completely new target locations in the present study. This lack of familiarity might have prevented relearning to occur [40].

When target relocations are unpredictable, observers only learn to associate one target location with a given repeated context. Hence, the current findings are incompatible with the view that the memory representations underlying contextual cueing can integrate up to two target locations [4], [7], [8]. If this view would apply unconditionally, repeated search for two target locations in one invariant context should enable learning of both locations. However, this was not observed in the present study, even when observers had more experience with relocated targets than with initial target locations.

Although single-target learning renders contextual cueing less flexible than previously proposed [8], it nevertheless permits rapid detection of at least one target location [10]. In fact, if two target locations were associated with one context, they would compete for focal attention [8]. Hence, the benefit deriving from contextual cueing would be reduced compared with learning a single target location [4], [8]. In contrast, single-target learning prevents competition between target locations. Taken together, the results of the present study suggest that besides repeated exposure further factors can modulate contextual cueing: While proactive interference seems to impede the successive acquisition of new memory representations with gradually fading impact, single-target learning [10] severely constrains the adaptation of established memory representations of context-target relations.

Unlike proactive interference, contextual cueing seems to be unaffected by retroactive interference. This is evidenced by a common observation throughout the present experiments, namely: the overall stability of contextual cueing for learned contextual associations following relocated targets and new-learning. Thus, the current results confirm that the retention of implicit contextual associations is not prone to temporal decay or effects of noise and of additional associations [5], [10], [15], [16], [31], [32], [41]. As proposed by Alberini (2011, [42]), consolidated memories typically remain stable when learning has reached an asymptotic level. Although contextual cueing may not have reached asymptotic levels in the present study (owing to the short learning phase), memory representations appeared to be robust enough for largely unaffected retention across fairly long periods. Furthermore, successful new-learning combined with contextual cueing in the return phase indicates that implicit (contextual) learning is based upon high, or even unlimited, capacity for at least distinct memory representations [16], [21], [43].

In conclusion, the present study shows that relearning of old contextual associations is inflexible in comparison to the successive learning of new contextual associations. We propose that the adaptive properties of relearning are restricted because a given context can only be associated with a single target location – which is likely to help minimize or avoid competition between multiple target locations [10]. At the same time, an existing association between a target location and a context is remarkably solid and durable, continuously facilitating efficient visual search across inconsistencies and long periods of time. Unlike relearning, in new-learning, context-target associations are not jeopardized by structural changes in the learned contexts. Hence, new-learning sets in once proactive interference subsides.
